# Supporting Family Caregivers’ Clinical Communication Skills: Adapting a Cancer Caregiver Communication Model for Dementia Caregiving

**DOI:** 10.3390/ijerph23020225

**Published:** 2026-02-10

**Authors:** Tyler S. Nesbit, Emma Bryan, Easton N. Wollney, Melissa J. Armstrong, Carma L. Bylund, Carla L. Fisher

**Affiliations:** 1 Department of Family, Youth and Community Sciences, College of Agricultural and Life Sciences, University of Florida, Gainesville, FL 32611, USA; tnesbit@ufl.edu; 2 Department of Psychology, College of Arts and Sciences, University of Miami, Coral Gables, FL 33146, USA; egb46@miami.edu; 3 Department of Health Outcomes and Biomedical Informatics, College of Medicine, University of Florida, Gainesville, FL 32611, USA; eastonwollney@ufl.edu (E.N.W.); carma.bylund@ufl.edu (C.L.B.); 4 Department of Neurology, College of Medicine, University of Florida, Gainesville, FL 32611, USA; melissa.armstrong@neurology.ufl.edu; 5 UF Health Norman Fixel Institute for Neurological Diseases, Gainesville, FL 32608, USA

**Keywords:** family caregiver, clinical communication strategies, dementia, caregiver-centered care

## Abstract

**Highlights:**

**Public health relevance—How does this work relate to a public health issue?**

- Family caregivers spend about 90 h weekly providing care, accounting for nearly USD 350 billion in care annually, with little to no psychosocial support.
- Clinical teams increasingly rely on dementia caregivers as dementia progresses and could benefit from clinical communication skills guidance.

**Public health significance—Why is this work of significance to public health?**

- The Caregiver Clinical Communication Process Model (C^3^PM) can guide caregivers on strategies to enact before, during, after, and between medical encounters to effectively communicate to achieve care goals.
- In dementia caregiving, emotionally focused communication is prioritized to achieve emotionally centered care goals like preserving the loved one’s dignity and buffering them from stress related to medical care.

**Public health implications—What are the key implications or messages for practitioners, policy makers and/or researchers in public health?**

- Dementia caregivers would benefit from psychosocial support that helps them build clinical communication skills.
- Nurses and clinical teams can utilize the C^3^PM-Dementia model as a visual tool. It can be provided to caregivers soon after diagnosis so that caregivers have a guide to refer to and utilize in their own time and as the disease progresses.

**Abstract:**

Background: Psychosocial support that enhances caregivers’ clinical communication skills can alleviate distress while enhancing their ability to communicate with nurses and clinical teams to achieve care goals. We sought to adapt a cancer caregiver clinical communication model (C^3^PM-Cancer) for dementia spousal caregivers that identifies key communication strategies they can enact before, during, after, and between appointments to promote better care. Methods: Interviews were conducted with caregivers of spouses diagnosed with dementia within the last 10 years. Data were thematically analyzed to confirm and extend the communication strategies and care goals in C^3^PM-Cancer to develop C^3^PM-Dementia. Results: Caregivers for spouses with dementia in our sample reported the same strategies and goals in each communication phase of C^3^PM-Cancer, which provides support for the utility of these caregiving communication skills across these two disease contexts. They described the importance of new communication strategies, which informed an emotionally focused communication approach used to protect their spouse’s personhood and dignity. The findings inform the adapted C^3^PM-Dementia. Conclusions: C^3^PM-Dementia can be an educational tool offered by clinicians to caregivers to provide guidance on key communication strategies to enact before, during, after, and between appointments to achieve critical care goals. The model can enhance communication between caregivers and clinicians, which can promote better outcomes in both cancer and dementia care.

## 1. Introduction

The U.S. population of older adults living with Alzheimer’s disease or related dementias (AD/ADRDs) is increasing in terms of both the number of individuals as well as the percentage of the population. Nearly 7 million people over the age of 65 are currently living with AD, the most common cause of dementia [1]. By 2060, this number is expected to nearly double [2]. The Alzheimer’s Association estimates that the percentage of deaths attributed to AD increased by 142% in the last two decades from 2002 to 2022, whereas other leading causes of death, such as heart disease, decreased [1].

The impacts of dementia are progressive, and there is no known cure for the disease. As dementia progresses, individuals will inevitably require increased support, including greater levels of assistance to complete activities of daily living as well as instrumental tasks [3]. Today, 83% of caregiving for older adults in the U.S. is performed by unpaid family members and friends, with an estimated 11 million of these unpaid caregivers providing care for AD/ADRD [1]. The Alzheimer’s Association estimated that in 2024 alone, unpaid caregivers provided more than 19 billion hours of care valued at USD 400 billion [1,4]. More than 60% of these caregivers are spouses or significant others [4]. Caregivers typically receive little to no psychosocial support. Yet, as the disease progresses, they play an increasingly central role in their loved one’s care both at home and in clinical settings and thus experience significant care burden. In the last years of life, spousal caregivers can provide between 43 and 90 h of care per week [5], with at least one study finding more than 20% of dementia caregivers providing care 24 h a day, 7 days a week [6]. In addition to ongoing financial challenges, dementia caregiving negatively affects family caregivers’ own health, with detrimental impacts on their physical, mental, and social health contributing to significant psychological distress, burden, and potentially burnout [6,7].

Psychosocial support that targets caregivers’ communication skill development can alleviate distress and burden and promote healthier outcomes while enhancing their caregiving ability [8,9,10,11,12,13,14]. While these interventions have primarily targeted cancer caregivers, this form of psychosocial caregiver support has been successful in alleviating caregivers’ distress and burden, as well as improving overall quality of life [8,9,10]. Communication interventions for caregivers of individuals with dementia typically focus on communication skills for managing communication deficits due to dementia progression. A scoping review found that this type of psychosocial communication support can improve both caregivers’ quality of life and the well-being of their loved one living with dementia [11]. Recent systematic reviews in dementia care provide strong evidence for communication-focused interventions as psychosocial support that improves caregivers’ communication knowledge, skills, attitudes, and self-efficacy, while reducing their stress, burnout, burden, distress, depression, and anxiety [12,13,14]. Ultimately, communication skills contribute to better psychosocial outcomes (e.g., improved relationships and less distress). Moreover, when caregivers receive such communication-based support, their loved ones living with dementia can experience reduced symptoms of depression, agitation, disorientation, irritability, and withdrawal [13]. Scientists suggested that these patient outcomes likely improved because caregivers themselves were communicating better, which in turn can improve relationships between caregiver family members and daily communication about care.

What has received considerably less attention, however, is caregivers’ clinical communication skills. Caregivers’ ability to communicate with their loved one’s clinicians (i.e., nurses, physicians, therapists, and social workers) is central to promoting quality dementia care and achieving critical caregiving goals. This lack of focus in communication support is particularly concerning as clinicians increasingly rely on caregivers’ communication during medical encounters given the inevitable cognitive decline of the person living with dementia [15]. Moreover, caregivers describe communication breakdowns and barriers during healthcare interactions and how these communication challenges inhibit their ability to have their caregiving and support goals met [16]. Thus, caregivers of individuals with dementia would benefit from clinical communication guidance or training that can support them in their role and promote better psychosocial outcomes.

We originally developed the *Healthy Communication Practice* online intervention for adult-child blood cancer caregivers to help them reduce distress by developing communication skills central to their caregiving role, with one part of the program helping caregivers develop skills for talking with their family member’s clinicians [8]. It was found to be feasible, acceptable, and effective in reducing distress over time by enhancing caregivers’ caregiving communication skills in several care domains, including clinical communication [8,17]. The program utilizes authentic caregiver narratives drawn from research along with evidence-based communication models of key communication strategies to teach caregivers the skills in a memorable and relatable way [18,19,20,21]. Given its impact, we have conducted formative research to adapt the intervention for the needs of caregivers of a spouse with blood cancer. Through that mixed-method research, we identified key skills they engage in to promote their ability to achieve critical care roles [17,21].

**Figure 1:**
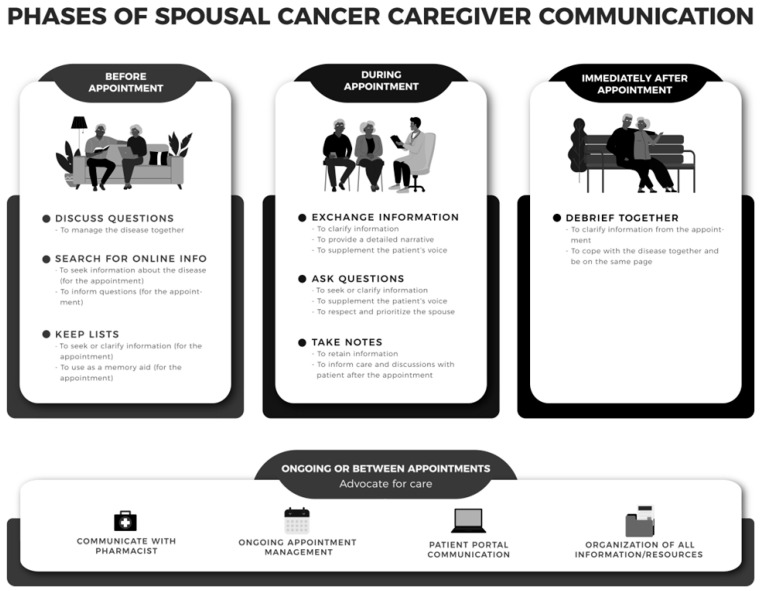
The Caregiver Clinical Communication Process Model for Cancer Caregivers (C^3^PM-Cancer)—Strategies and Associated Goals (Reprinted with permission from Ref. [21]. 2024, Wolters Kluwer Health, Inc.).

These findings informed the development of the Caregiver Clinical Communication Process Model (C^3^PM-Cancer), which provides a unique caregiver-centric approach to clinical communication and addresses caregivers’ skill needs before, during, and after clinical encounters to achieve care-related goals (see Figure 1). We initially developed this model in our caregiving research with spouses caring for blood cancer patients [21,22,23]. The model highlights the importance of viewing clinical communication as a communication process (i.e., phases of communication) as opposed to only focusing on communication with clinicians during appointments (i.e., one phase of communication).

For instance, our formative work to develop the model showed that caregivers’ ability to communicate with clinicians during appointments is informed by skills they enact before appointments (in preparation for clinical communication) and after appointments (to debrief with their loved one about the appointment and the care plan), as well as between medical visits (to advocate for ongoing care with the clinical team) [21]. For example, caregivers enact a strategy of discussing questions with their spouse before appointments to engage in care together and collectively decide what they want to discuss in an upcoming appointment. Caregivers also enact the strategy of exchanging information during appointments to ensure that the clinician has all the details needed to promote quality care. And, after appointments, caregivers’ strategy of debriefing with their spouse is key to ensuring that they are on the same page.

Follow-up research on the model confirmed that caregivers perceive that the communication strategies in each phase of C^3^PM are highly relevant to their role. At the same time, caregivers reported that the strategies were not easy to learn and implement, demonstrating a need for communication skills training/support to help caregivers develop these communication competencies [17].

To adapt *Healthy Communication Practice* to best serve the needs of dementia spousal caregivers, we first aimed to confirm that the communication strategies and goals represented in C^3^PM-Cancer are relevant to dementia caregiving and related clinical communication. Additionally, although blood cancer care likely shares similarities with dementia care as they can both be chronic conditions, dementia care has complex characteristics that are embedded within a unique disease trajectory. There is no cure, and patients will ultimately face ongoing, progressive cognitive decline resulting in increasing caregiver dependence. This type of disease progression contributes to significant caregiving burden, particularly in the healthcare setting, as clinicians increasingly rely on the caregiver’s communication to manage dementia care. Therefore, caregivers’ ability to engage their loved one with dementia in medical interactions is further challenged and undoubtedly evolves uniquely in dementia caregiving. Thus, in addition to confirming the utility of the model for caregivers of spouses with dementia, we also aimed to adapt the model based on caregivers’ distinct experiences to create C^3^PM-Dementia to best serve their unique caregiving needs.

## 2. Materials and Methods

To prioritize the stakeholders’ voices in adapting the model, we used a qualitative design with deductive and inductive analyses to both confirm the strategies and goals of the C^3^PM-Cancer model in dementia caregiving and extend the model by adapting it to target dementia caregivers’ needs and created C^3^PM-Dementia. A multidisciplinary team of experts in clinical and family communication, caregiving, neurology, and gerontology, as well as qualitative methodologies in intervention development, promoted an interdisciplinary approach central to the study aims.

To be eligible, participants were required to have (1) cared for a living/deceased spouse diagnosed with AD/ADRD within the last 10 years, (2) be English-speaking, and (3) reside in Florida. The Florida-based inclusion criterion was a requirement of the funding agency. Purposive sampling and a multi-channel approach were employed to recruit caregivers using the following means: (1) posting on the Alzheimer’s Association clinical trials registry; (2) emails and flyers disseminated to community organizations such as non-profit dementia caregiving service providers, adult day care centers, resource fairs, caregiver conferences, caregiver support groups, and faith-based organizations; and (3) emails shared with dementia providers at UF Health Memory Clinic and related listservs. No channel involved direct recruitment by staff with potential participants with whom they had established relationships/associations to prevent coercion. Caregivers receiving study information (e.g., via email, disseminated flyer, or web posting) who were interested could inquire about the study by emailing our research staff only, using an email address provided in the study flyers. If they met our research staff at a community event, they had the option to screen/enroll or take study information home to decide. Two recruited participants learned about the study through team members’ social networks (those team members did not enroll or engage with them as research participants, and the participants contacted a research team member independently). Interested participants were screened for eligibility via phone with a study team member (E.B. and T.S.N.) and to schedule an interview. Once enrolled, participants were asked to complete a brief online survey. This included a waiver of written informed consent consistent with the IRB exemption (ET00021681), outlined the purpose and procedures of the study, anticipated risks and benefits, and provided participant compensation information (USD 50 Amazon e-gift card). Demographics were also obtained in this survey.

In-depth, semi-structured interviews were audio-recorded and conducted via the Zoom videoconferencing platform or telephone. An interview guide used with different cancer caregiver types in the development of *Healthy Communication Practice* [8,21,24,25] was modified in its language for dementia caregiving (i.e., to reference dementia or spousal relationship as opposed to cancer) by team members with clinical and scientific expertise, including communication/health behavior scientists (C.L.F, C.L.B, and E.N.W.), an expert in clinical social work (C.L.F.), and a neurologist (M.J.A.). The team has expertise in family caregiving and dementia (C.L.F., C.L.B., M.J.A., and E.N.W.), neurology and dementia care (M.J.A.), and caregiving and clinical communication (C.L.F., C.L.B., M.J.A., and E.N.W), and all teams members have previous qualitative methodology experience and expertise. Caregivers were asked about their communication experiences in clinical and family settings relevant to dementia caregiving. To address the model, the guide was centered around each phase of the communication process (e.g., before, during, and after appointment). We began with an inductive approach by allowing caregivers to openly share (on their own) any communication strategies they used in that communication phase (e.g., before appointments) and perceived to be relevant to clinical communication, as well as their motivations (goals) associated with the strategy (e.g., [before appointments] “Do you do anything to prepare for medical appointments?” [followed by probes]). This inductive approach ensured we could identify distinct approaches to dementia caregiving by allowing caregivers to naturally report communication strategies/goals that we may have (or not) previously identified in each phase of the model (as opposed to assuming they enacted the strategy). Caregivers were then asked, using a deductive approach based on the model of strategies (themes) and associated goals (thematic properties), about their use of each strategy (e.g., keeping lists) and associated goals (e.g., to see or clarify information during the upcoming appointment) in each communication phase (e.g., [if not mentioned] “Do you keep lists of what you want to address with the clinician? Why/why not?” [followed by probes]). Two team members with previous qualitative methodology experience (E.B. and T.S.N.) conducted all interviews after intensive training by the senior author (C.L.F.). Interviews were on average 93 min (range: 61–120 min). Professional transcriptions resulted in 416 single-spaced pages of data.

Transcripts were thematically analyzed using the widely utilized constant comparative method approach including both deductive and inductive analyses [26,27]. The qualitative data analysis software ATLAS.ti 25.0.1 (Scientific Software Development GmbH, (Denver, CO, USA)) was used to manage the data [28]. A rapid analysis was conducted concurrently with data collection to promote thematic saturation and deductively track caregivers’ use of communication strategies from C^3^PM-Cancer. In other words, we deductively analyzed the data for the previously established typology of strategies identified in the original model. An inductive analysis was performed concurrently to ensure that new strategies (not pre-determined or informed by theory) could be identified to illustrate distinct needs in dementia caregiving. This also allowed for a confirmation of the model in dementia caregiving while capturing novel findings. By doing this rapidly during data collection, we were also able to develop a preliminary codebook for an in-depth thematic analysis once all interviews were complete. We continued using the best practice of combining deductive and inductive analyses for this in-depth analysis of the data. The analysis was led by two coders with previous qualitative analysis experience (E.B. and T.S.N.) and overseen by the senior author, an expert in thematic analysis and qualitative design (C.L.F), who also provided oversight across the analytic process as an additional coder. We used a consensus approach to develop a codebook, which was guided by and overseen by the senior author, who is also an expert in the content of the data (caregiving communication). Multiple meetings were held between the coders and with the senior analyst (C.L.F) to address analyses and come to a consensus. The analysis identified caregiver communication strategies as themes organized by phase (e.g., before, during, after, or between appointments) consistent with C^3^PM-Cancer. Inductive analysis was initiated by a process of open coding to identify patterns that were not initially present in C^3^PM-Cancer. These patterns were collapsed into themes and then axially coded for patterns to further define each theme with thematic properties representing caregivers’ motivation (i.e., goal) for enacting a strategy. Owen’s established criteria for thematic saturation were used [29]. We employed multiple best practices in line with leading qualitative scholars utilizing interpretive designs that prioritize bringing the insider’s voice (i.e., participants) to the forefront of the findings. We used Morse et al.’s verification techniques to ensure rigor across the design process (e.g., purposive sampling and methodological congruence), including during analysis (e.g., multiple coders, content and methods experts, expert overseeing analysis, and inductive and deductive analyses) [30]. The extent of thematic saturation ranged from 13 to 100% (an average of 60%, or at least 9 out of 15 caregivers), with most strategies in the model reported by more than half of caregivers.

## 3. Results

Fifteen spousal caregivers participated. On average, caregivers were 72.2 years old (range: 49–87), with most identifying as White (87%). The majority were female (67%). All but two caregivers had at least one child, and over 73% were retired. The caregivers’ diagnosed spouse was on average 77 years old (range: 64–90). Additional demographics are presented in Table 1.

The findings helped to confirm the utility of the model and extend it by adapting it to meet the needs of caregivers of a spouse living with dementia. Caregivers of spouses with dementia in our sample reported using all of the same strategies (with associated goals) identified in C^3^PM-Cancer in all four phases. Thus, the findings suggest the utility of the communication process model for caregivers of a spouse with dementia, providing initial findings that support the transferability of the model for dementia caregiving.

Caregivers also described slight differences with two strategies (e.g., before–discuss questions; after–debrief together). These differences were informed by distinct needs in dementia caregiving further discussed below. Additionally, caregivers identified four new strategies enacted before, during, and after appointments, or across the communication process (before–provide reminders and explanations; during–express emotional support and promote patient engagement; after–engage in a relational ritual). All of these new strategies addressed emotional care needs related to reducing distress for their spouse with dementia. Collectively, these strategies encompass an overarching emotionally focused communication approach enacted in dementia caregiving to achieve emotionally centered care goals (e.g., to provide reassurance to reduce the spouse’s distress). This new finding demonstrates how dementia caregivers prioritize emotionally focused care in dementia caregiving.

**Figure 2:**
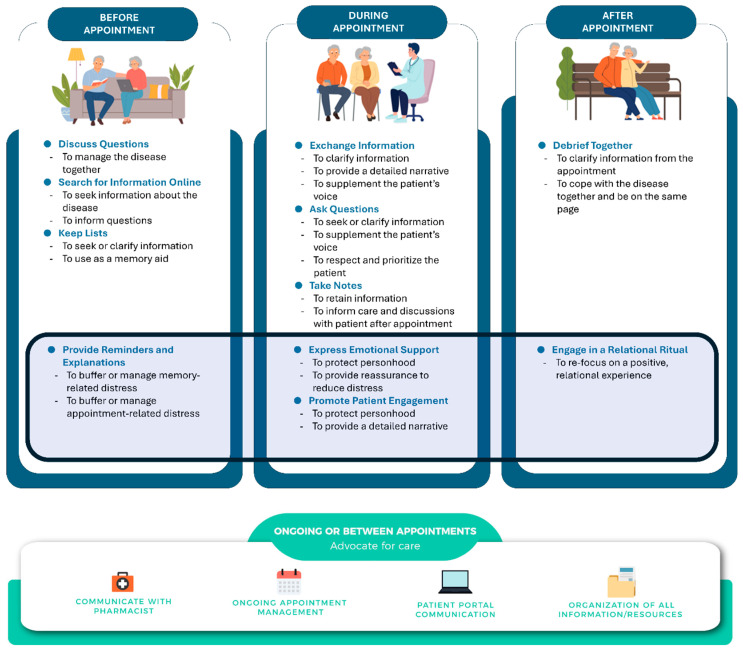
The Caregiver Clinical Communication Process Model—Dementia (C^3^PM-Dementia). Note: The shaded box includes new strategies and associated goals dementia caregivers utilize. (Adapted with permission from Ref. [21]. 2024, Wolters Kluwer Health, Inc.).

Caregivers’ use of strategies (i.e., themes), both those that were confirmed from the original model and new strategies that emerged, are presented below as subheadings according to each phase of the communication process in the model. Results include any differences in how caregivers of spouses with dementia described the strategies to prioritize their spouses’ distinct needs related to dementia care. In line with the original model (C^3^PM-Cancer), for each strategy, the associated caregiving goals (i.e., thematic properties) are presented in italics and illustrated using evidence or caregivers’ narrative accounts. See Figure 2 for the adapted C^3^PM-Dementia (with newly identified strategies highlighted within a box in the original model), and see Table 2 for a full typology of findings to promote the translation of findings to practice.

### 3.1. Before the Appointment

Dementia caregivers confirmed their use of the three strategies enacted before the appointment in C^3^PM-Cancer: discuss questions, search for information online, and keep lists. They also identified a fourth strategy distinct to dementia caregiving: provide reminders and explanations.

#### 3.1.1. Discuss Questions

Discussing questions before appointments was described by dementia caregivers slightly differently than it was by cancer caregivers. Like cancer caregiving, dementia caregivers identified the same goal in that they discussed questions with their spouse prior to the appointment *to manage the disease together*:

On the way to the appointment, I’ll say, “okay, we’re headed to the appointment. Is there anything you … would like to discuss so we won’t forget and we’ll write it down?” And most of the time, he’ll say no. But like the last time, he said, “well, the only thing different that I’ve noticed is that I have a little numbness in my feet.” (wife, age 51)

However, this strategy was less focused on by dementia caregivers than cancer caregivers, and their communication was described more as information sharing rather than collaborative communication. This difference was related to their spouse’s ongoing cognitive decline: “I think it’s just a lot for him to think and understand really what’s going on. I mean, I try to be clear with him, and I say the questions in front of him. So, he knows the questions” (wife, age 73).

#### 3.1.2. Search for Information Online

Dementia caregivers looked for information online before appointments in line with the previously identified goals in C^3^PM-Cancer. First, they wanted *to seek information about the disease*:

That’s what I did when it came to that particular blood work that they did. I did go online and look at what it talks about. So, I do a lot of research online for different tests or even when they do medications to make sure I’m more informed. (wife, age 49)

Online information was also used *to inform questions for clinicians*, particularly regarding symptoms they observed in their spouse:

If she was experiencing some symptoms, I would try to get some idea ahead of time of whether it might be this medication or that medication, and then when we would see the doctor, I would say, “Do you think this is because of this?” And sometimes it was and sometimes it was not. (husband, age 87)

#### 3.1.3. Keep Lists

Dementia caregivers identified the same two goals as cancer caregivers did when keeping lists. Lists were kept *to seek or clarify information*. They described doing this to address their spouse’s behaviors during appointments to seek further guidance:

I started keeping a log of behaviors that I saw. … Without that log, it would have been difficult for me to describe anything. … So, I tried to go in with bullet points. Boom, boom, boom. This is what’s happening, and here are my questions. (wife, age 77)

Additionally, caregivers kept lists prior to appointments *to use as a memory aid*: “I usually write down my questions, so I won’t forget anything … between appointments. Like, if he had an appointment today and the next one is in a month, I usually keep track of things within that time” (wife, age 51).

#### 3.1.4. Provide Reminders and Explanations

Dementia caregivers identified a new strategy that centered on priming their spouse for upcoming appointments to prioritize their emotional well-being. Ultimately, they sought to emotionally prepare their spouse to reduce two inter-related forms of distress. Caregivers both reminded their spouse and provided explanations about upcoming appointments *to buffer or manage memory-related distress*. Caregivers explained that because their spouse had memory challenges, they typically would not recall upcoming appointments or critical details like the reason for the medical visit or who the provider was. This created distress for their spouse. Reminders helped prepare their spouse and protect their emotional state:

[I say] “You have a doctor appointment.” [She asks,] “Well, what kind of doctor is it?” [I say], “It’s a neurologist.” [She responds], “Well, why am I going to see a neurologist?” [I explain]. “Because he manages your medication.” … Sometimes it would be, “Well, you like this doctor, you trust this doctor. She’s very kind, and she tells you everything you want to know. And she listens to you.” [My wife will say], “Oh yeah.” Whether she actually did [recall] or not, I don’t know. But it was basically trying to help her understand the context and come to the same conclusion that I was coming to. (husband, age 87)

Additionally, caregivers shared that reminders/explanations were needed *to buffer or manage appointment-related distress.* Caregivers shared that their spouses were distressed about the appointment, which informed their resistance or “apprehension” to going to the appointment. As such, reminders and explanations were critical to alleviating some distress or helping to “calm that down” (husband, age 80). As another caregiver explained: “I try to tell him everything about the appointment because he gets anxious. … I try to stay light, a little bit, because he can’t manage me not being positive” (wife, age 77). At times, caregivers stressed the importance of keeping reminders/explanations short and directive to prevent their spouse’s distress from increasing:

I would say “Look, let’s just go,” and basically go from there. I would try to … minimize the communication so I didn’t give him room to get more frustrated and more worked up about it. So, one of the techniques is to kind of walk away, let him cool off and then try again. So, in that sense, yeah, just try to work the best you can. (wife, age 67)

### 3.2. During the Appointment

During appointments, caregivers used the same three strategies identified in C^3^PM-Cancer: exchange information, ask questions, and take notes. Caregivers also reported using two additional strategies distinct to dementia caregiving: express emotional support and promote patient engagement.

#### 3.2.1. Exchange Information

Dementia caregivers described the same three goals associated with exchanging information as cancer caregivers, while stressing the importance of doing so in a way that respected and prioritized their spouse’s voice. They explained that it was important to exchange information with clinicians *to clarify information* related to their ability to fulfill their caregiving role in helping their spouse manage symptoms and manage their future care needs: “If I’m noticing there are some changes in [his] behaviors, then I’m informing [the clinicians] of that and trying to figure out what’s the game plan going forward” (wife, age 49). Caregivers also exchanged information *to provide a detailed narrative* for clinicians, which was key to obtaining quality care: “I just wanted to be damn sure that I was able to provide all the information that I could in case there was something that was unique or different [about my wife’s disease]” (husband, age 80). Caregivers shared that they learned the importance of exchanging information to achieve this goal in a respectful manner:

I would start off with something like, “Well, I can fill in some of the things that [my husband] is saying. I can fill in with more information.” So, I wasn’t saying “No, you’re wrong” or anything like that or minimizing his involvement, but I was kind of adding to it. So, that felt better, especially because initially, there was more of that confrontational thing—he says, she says type of thing. With leading in saying, “Let me fill you in with what he was saying,” or “Let me give you an example.” (wife, age 67)

Lastly, caregivers needed to share information *to supplement the patient’s voice,* which was critical when their spouse would not (or could not) fully answer the clinician: “I make sure that [wife] is the one that is the focus of the conversation and she can tell us. And I just fill in here and there when I need to [like] when she’s forgetting something [not on purpose]” (husband, age 76). Caregivers also described learning how to do this in a way that still prioritized and respected their spouse’s voice, which could coincide with providing a detailed narrative:

I’m a lot more laid-back [now]. I wait, and a lot of times now he will defer to me. But I’m not as worried about what he’s going to say or what the doctor’s going to say. I’m just a lot more casual about it to relax the setting. … I just go with the flow now. If it isn’t a big deal, I don’t even answer for him. Like if he said, “Eggs … eat them every day,” knowing that he hates eggs now, I wouldn’t say, “No, you don’t!” [laughs] But if it’s something important medically, then I have to do something about that. (wife, age 77)

#### 3.2.2. Ask Questions

Dementia caregivers confirmed that they asked questions during appointments for the same three previously identified goals in C^3^PM-Cancer. They asked questions *to seek or clarify information* related to their spouse’s care: “I always ask questions [laughs]. Yeah, I mean, I try to make them relevant to our goal…I ask questions about anything I think is relevant, that can make it better for him” (wife, age 77). Caregivers also illustrated how they asked questions *to supplement the patient’s voice*. This goal seemed to become increasingly important as the disease progressed: “I mean, I would ask all questions … essentially almost functioning as a guardian or parent-child kind of relationship” (husband, age 67). Finally, caregivers asked questions during appointments *to respect and prioritize the patient*. This emotionally focused goal was central to their caregiving role in the moment and could work in tandem with the other goals (e.g., supplementing the patient’s voice):

When we were there, and this is, again, something that can be difficult to do when you’re a caregiver, but when we were there, I let him articulate to the best of his ability how he was feeling. [The doctor] very professionally addressed him, and not me. … Towards the end of the appointment, I would say, “may I share some of the things that I have observed over the past 90 days?” (wife, age 66)

#### 3.2.3. Take Notes

Dementia caregivers also reported the same goals for taking notes during clinical encounters as cancer caregivers. Notetaking supported caregivers’ ability *to retain information,* which supported their own memory: “I’m not young. I’ve got so many different things going on with [my husband] that I can’t remember all of them and his medications and what they’re for” (wife, age 73). Caregivers also took notes *to inform care and discussions with the patient after the appointment*. This gave caregivers an opportunity to address something that they learned during the appointment later or in the future: “It might be that there is a clinical study. … So, [the clinician] brought that up and we thought it was a good idea” (husband, age 76).

#### 3.2.4. Express Emotional Support

Dementia caregivers identified a new strategy central to dementia caregiving by describing the importance of expressing emotional support verbally and nonverbally to promote or protect their spouse’s psychological well-being during the clinical visit. For instance, they engaged in emotional support *to protect the personhood* of their spouse, which promoted their emotional well-being by reducing their vulnerability and prioritizing their dignity. Caregivers stressed that it was important to use a positive tone, to speak lovingly, to reframe things from their perspective, and to not directly correct their spouse. This emotionally supportive communication was particularly important during discussions with clinicians about their spouse’s safety and loss of independence:

[My husband] said, “I drive just fine.” And I said, “I know, honey. I know you drive just fine. But there are a lot of people on the road that don’t. And I’m worried that your reactions are not quick enough.” And he couldn’t argue with that, you know. I would say, you know, “Yeah, you drive fine. I know you can drive fine. But your reactions have slowed down. And if somebody makes a mistake, not you, but if somebody else makes a mistake and it happens every day, I’m not sure you could catch it quick enough to keep from having an accident.” (wife, age 71)

Caregivers also explained that expressing emotional support was important when appointments became upsetting to their spouse and that it could help to express gratitude for being involved: “I always thank him. … I never take it for granted. I always say, ‘Thank you for allowing me to help you or assist you’” (wife, age 67). Caregivers also expressed emotional support *to provide reassurance to reduce distress* that arose during the appointment, which could happen when providers brought up difficult care topics (e.g., long-term care):

[I was] very quick to say, “That’s not going to happen. We’re in this together, we’ve been married for X number of years, and we work through these things together, so that’s not going to happen.” I would just say, in front of my wife, “That won’t happen. You don’t need to worry about that.” I reassure her, with the doctor there, of course, to hear it. (husband, age 67)

Caregivers also enacted this strategy to reassure their spouse about clinical procedures that needed to be performed that their spouse found distressing:

[My husband] would say to [the doctor], “Who walks up to you and repeats five words, and then tells you to repeat them back?” Like [as if to say], “This stuff is stupid.” And of course, I put my hand on [my husband’s] arm and I’d say, “Honey, it’s all part of the process. Not to worry.” Again, when you have a close personal relationship like a marriage, he would take that and say, “Oh, yeah, yeah, you’re right.” And then it’d be over. (wife, age 66)

#### 3.2.5. Promote Patient Engagement

Another new strategy caregivers engaged in was promoting their spouses’ engagement during clinical encounters. Caregivers did this to achieve the same goal as emotionally supportive communication: *to protect personhood.* This goal seemed especially pertinent to living with dementia as caregivers wanted to protect their spouse’s identity and emotional state.

I try and leave it up to him, to give him his independence. … I don’t want him to feel like he’s less than he was. I don’t want to take away his masculinity, …to make him feel that he’s not able to function. (wife, age 81)

Caregivers highlighted how by engaging their spouse it prioritized their independence: “I’m very careful not to dominate the conversation. So, the things that [she] can discuss, even if she can’t find the word right away, I’m really careful not to talk for her” (husband, age 76). Caregivers also tried to promote their spouse’s involvement in the clinical interaction *to provide a detailed narrative.* Caregivers shared that they would look to their spouse to confirm information and ask them directly to engage them:

Going to the psychiatrist and [the psychiatrist asks her], “Well, how do you feel?” and she says, “Fine.” And I said, “You know, you really haven’t-- you’ve been upset a lot.” … And sometimes she would verbalize more about it, “I really feel anxious, I don’t know what’s going on.” And just in general would expand a little more on the subject, even though initially, she had blown it off. (husband, age 87)

### 3.3. After the Appointment

After appointments, caregivers used the same strategy identified in C^3^PM-Cancer: debrief together. They also described an additional strategy focused on the emotional and relational well-being of their spouse: engage in a relational ritual.

#### 3.3.1. Debrief Together

Caregivers confirmed the importance of debriefing about the clinical encounter together and identified the same two goals motivating this behavior. Caregivers used these debriefing sessions to clarify information from the appointment. Their approach was slightly different than cancer caregivers’ in that their spouse’s cognitive decline contributed to discrepancies in recalling what happened during appointments. Caregivers described adapting their approach to address this challenge:

What I do is actually a little bit different. I cheat. We talk on the phone with his daughters on the speaker. So, he hears me. He hears me repeating kind of what went on, what was said … He has also swallow problems, for example. … So, if he doesn’t want to discuss it, well, guess what? It’s something that has to be discussed. … I realized “Well, hold on, this is a pretty good way of making sure he hears the feedback too or understands the reality or maybe the seriousness of the condition or understands more about it.” (wife, age 67)

Additionally, caregivers described how they would debrief to cope with the disease together and be on the same page. This too could be challenging given their spouse’s memory decline; however, caregivers still stressed the importance of communally coping together:

I just go over everything that we touched on and what they said. I had to be honest because he thinks [sic] he was going to get better and he’s not. I don’t dwell on that, but if it comes up, then I say “Remember the diagnosis. We have to work with that.” (wife, age 77)

#### 3.3.2. Engage in a Relational Ritual

Caregivers also engaged in a relational ritual immediately following the appointment, a strategy unique to dementia caregiving. This ritual was a relational activity their spouse enjoyed such as going for ice cream, having lunch with loved ones, or going to church. Caregivers described the importance of having a ritual after the appointment *to re-focus on a positive, relational experience*. This strategy was critical to reducing or managing their spouse’s distress from the appointment and the reality of living with dementia:

I try to do something not medical. I try to take him out of that mindset, like go to a nice lunch, or call my son-in-law and see if he wants to go to lunch with me, with us… to refocus us, I guess, away from the diagnosis and the problems and the medicine and all that. (wife, age 77)

### 3.4. Ongoing Communication or Between Appointments

In line with the original model, caregivers described engaging in the same strategy and associated goals between appointments: advocate for care.

#### Advocate for Care

Caregivers confirmed the importance of advocating for their spouses *to ensure quality care* between appointments. This included needing to address medication issues or behavioral changes in between appointments:

He was sleeping all the time. And I thought, “you know, this isn’t like him to be sleeping all the time.” So I read about each one of his medicines and each one of them, … there is a component of drowsiness. … He was taking four medications and three of them were eliminated. And I stuck with the blood pressure. And the other three had been prescribed years ago. (wife, age 83)

Caregivers also advocated for their spouse in order *to coordinate care*. This involved managing communication with multiple providers to address needs or scheduling for appointments, medications, and procedures. They described communicating through their spouse’s electronic chart to discuss their spouse’s care with their healthcare team:

I do a lot on the MyChart ©. Different [doctors] and having to do with vaccinations and immunization type things, when to do those. [If I] observe something that looks a little weird, I’ll generally drop a note on that. I try to keep them up to date in terms of where she’s at in terms of progression, at least as I see it. (husband, age 79)

## 4. Discussion

Collectively, the findings provide guidance for caregivers of a spouse with dementia to enhance their clinical caregiving communication. In confirming the importance of the previously identified communication strategies and associated care goals for all phases represented in C^3^PM-Cancer, we demonstrate the utility of the model in another disease caregiving context—dementia care. We also extend our understanding of dementia caregivers’ distinct clinical communication needs that are relevant to the challenges of caring for a spouse with dementia. In creating C^3^PM-Dementia, we offer a tool that nurses and other clinicians can share with caregivers, soon after diagnosis, to enhance their ability to communicate with clinicians and achieve care goals. This model can also be implemented in a communication skills intervention to promote caregivers’ clinical communication competence. By receiving such communication skills support, this could enhance spousal caregivers’ psychological well-being while promoting their ability to achieve dementia care goals, including those that address their spouse’s emotional state.

It is especially noteworthy that the findings help illuminate the importance of emotionally focused goals in dementia caregiving, including how clinicians talk with caregivers during appointments. Caregivers consistently described how they prioritize their spouse’s emotional well-being across every phase of the clinical communication process. They enacted strategies to achieve emotionally focused goals, thereby demonstrating the importance of prioritizing the socioemotional health of individuals living with dementia. Based on this, we also identified new strategies dementia spousal caregivers utilize to promote their spouse’s mental well-being, such as providing reminders and explanations before appointments, expressing emotional support during appointments, and engaging in a relational ritual after appointments. These communication strategies encompass an approach that can help caregivers alleviate, buffer, or manage their spouse’s distress before, during, and after clinical encounters. Thus, communication skills interventions for caregivers that utilize C^3^PM-Dementia may simultaneously promote better psychological well-being and adjustment for individuals living with dementia, like other communication-focused dementia interventions [13]. Further, the model itself can promote clinicians’ awareness of the importance of caregivers’ enactment of this communication approach and promote clinicians’ ability to communicate with caregivers and patients in ways that protect the dignity of those living with dementia.

Given the ongoing cognitive decline (and lack of cure) associated with dementia, the emotional well-being of individuals living with this disease is especially critical. There is a wealth of evidence informed by socioemotional selectivity theory to support this, demonstrating that as one’s perspective of time left in life decreases (as it does with aging and life-threatening diseases), present well-being and emotionally focused goals are prioritized as most critical to promoting a higher quality of life over future-focused goals/well-being (like gaining knowledge) [31,32,33,34,35,36]. While our study provides support for caregivers’ prioritization of their spouse’s emotionally focused well-being in dementia care, research on individuals living with dementia also shows that, regardless of living with a disease that increasingly distorts one’s sense of time, the evidence informing socioemotional selectivity theory holds true: older adults living with dementia continue to be motivated to achieve emotionally focused goals or experiences and prioritize their emotional well-being [37]. A focus on the emotional care of individuals living with dementia is further warranted given that living with dementia is associated with more psychiatric distress in comparison to typical aging in older adults [33,38].

Caregivers demonstrated their prioritization of emotionally focused goals across the clinical care and communication process and emphasized the importance of protecting their spouse’s personhood (i.e., what makes an individual who they are) and dignity (i.e., every person has worth–value). This is critical when interacting with clinicians. Personhood has been referred to as the “cornerstone” of person-centered dementia care as dementia not only creates challenges for self-expression and communicating one’s identity, but dementia caregiving specifically can be perceived as oppressive given patients’ loss of autonomy coupled with stigmatizing societal views of individuals living with dementia [39]. Caregivers in our study shared how they needed to advocate during clinical encounters to protect their spouse’s personhood and dignity during conversations with clinicians. A recent review demonstrated that the communication skills of both caregivers and clinicians are key to protecting the personhood of those living with dementia [12]. Moreover, patient engagement in the clinical conversation (as opposed to ignoring or always talking for their loved one) can continue to be a key strategy in protecting emotionally focused goals in care (i.e., reducing distress and protecting personhood) even as the disease impairs one’s cognitive ability to engage in higher-level discussions or care goals (e.g., decision making).

With this goal in mind, the adapted C^3^PM-Dementia can be integrated into existing interventions, like *Healthy Communication Practice*, or nurse-led education interventions that aim to teach caregivers these key clinical communication skills that are also tied to promoting the care and quality of life of their loved one. Dementia family caregivers not only lack, but yearn for caregiver support and education, often looking to nurses for support and guidance [40]. Moreover, psychosocial support in the form of communication skill development for caregivers is tied to enhanced health outcomes for caregivers (e.g., less distress and burden) [11]. Thus, future studies that integrate C^3^PM-Dementia in an intervention can investigate the impact for caregivers as well as its extension to improving the well-being of individuals living with dementia. While communication skill development is centered on strategies to enhance the emotional well-being of their spouse with dementia, caregiver–patient outcomes are inter-related [41], further suggesting that patients too will benefit from caregiver-focused communication interventions as well as communication guidance from their loved one’s clinical team. Future studies could also further explore how the strategies within each phase may be more prioritized at different phases of care depending on the progression of the disease impairing their spouse’s cognitive abilities and engagement.

C^3^PM-Dementia ultimately provides spousal caregivers with guidance on key communication strategies they can enact to achieve critical emotionally focused care goals during clinical encounters by enacting communication skills before, during, after, and in between appointments. Given this, the model can be translated directly to practice in more immediate ways, which may not only improve caregivers’ access to support but also extend to clinical education for non-specialists, which is also direly needed [40]. For instance, C^3^PM-Dementia, specifically the visual, can become a tool offered to caregivers in clinical settings (e.g., disseminated by nurses, social workers, case managers, neurologists, psychiatrists, and psychiatric nurse practitioners). Spousal caregivers can use the visual of the model as a tangible guide that they can then refer to and utilize in their own time and as the need for the communication strategies increases with disease progression. At the same time, the model can help nurses and other clinicians understand the importance of caregiver communication during medical encounters in promoting the emotional wellness of individuals living with dementia. In doing so, they can better identify and appreciate the type of communication caregivers are engaging in to protect their spouse with dementia and achieve care goals.

This study largely represents white, non-Hispanic caregiving wives in heterosexual relationships. The intentional inclusion of caregiving husbands is needed, and studies that use more strategic messaging may promote researchers’ ability to oversample caregiving husbands’ perspectives to ensure representation [42]. Additionally, to promote health equity and reduce disparities, a more diverse sample in terms of race, ethnicity, and sexual orientation is warranted. This research is needed to enrich the findings to identify overlap and distinctions in communication skill needs related to sociocultural diversity. Narrowing in on the type of dementia may also be appropriate given their variant trajectories and associated care needs [3,40]. Moreover, the severity or stage of dementia can also play a role in caregivers’ perceptions of the importance of the strategies. Thus, this model would also benefit from quantitative studies, similar to the process undertaken with the original version (C^3^PM-Cancer), by confirming the strategies with a larger sample of spousal caregivers that allows for comparison in experiences related to dementia type and severity [17]. Additionally, using a multi-method qualitative design, such as direct observation in multiple environments (e.g., at home and in clinic), triangulated with an interview methodology could further the findings by offering an opportunity for triangulating rich data, which would also further reduce the potential for bias.

## 5. Conclusions

The findings demonstrated substantial overlap with cancer caregivers’ reported strategies and associated care goals in the C^3^PM-Cancer model, demonstrating the transferability of the model to dementia caregiving. Spousal caregivers of a loved one with a dementia diagnosis are an integral part of dementia care and clinical communication. Their caregiving role is associated with increasing burden and distress as dementia progresses, and clinicians increasingly rely on communication with caregivers to promote quality dementia care. Communication skill development can provide caregivers with psychosocial support to promote better health outcomes and enhance their caregiving ability. Nurses and clinicians can use C^3^PM-Dementia to provide spousal caregivers with guidance on communication strategies they can enact to achieve critical care goals before, during, after, and in between appointments. These communication strategies can help caregivers promote quality care for their diagnosed spouse, enhance their ability to manage their spouse’s emotional distress, and improve caregiver–clinician communication, while potentially promoting better socioemotional health for caregivers and their spouse with dementia.

## Figures and Tables

**Table 1:** Participant Demographics.

	M	SD	Min–Max	
Age				
Caregiver Age	72.2	10.9	49–87	
Patient Age *	77.2	7.3	64–90	
Number of Children	1.7	1.2	0–5	
			**% (n)**	
Sex (Caregiver)				
Female			67% (10)	
Male			33% (5)	
Race/Ethnicity (Caregiver)				
White, non-Hispanic			87% (13)	
Black, non-Hispanic			13% (2)	
Length of Marriage (Years)				
5–10			7% (1)	
11–20			13% (2)	
20+			80% (12)	
Employment Status (Caregiver)				
Retired			73% (11)	
Full-time employment			27% (4)	
Annual Income (USD) (Caregiver)				
Prefer not to say			7% (1)	
0–29,999			13% (2)	
30,000–59,999			20% (3)	
60,000–89,999			20% (3)	
90,000–119,999			33% (5)	
120,000+			7% (1)	
Patient Diagnosis				
Alzheimer’s disease dementia			40% (6)	
Dementia unspecified			33% (5)	
Dementia with Lewy bodies			20% (3)	
Vascular dementia			7% (1)	

* Two deceased patients excluded from age statistics.

**Table 2:** Spousal caregivers’ communication strategies to achieve dementia care goals.

Spousal Caregivers Use These Strategies	to Achieve These Dementia Care Goals
**Before the appointment**	
Discuss questions	To manage the disease together
Keep lists	To seek or clarify information
	To use as a memory aid
Search for information online	To seek information about the disease
	To inform questions for clinicians
* Provide reminders and explanations	** To buffer or manage memory-related distress
	** To buffer or manage appointment-related distress
**During the appointment**	
Exchange information	To clarify information
	To provide a detailed narrative
	To supplement the patient’s voice
Ask questions	To seek or clarify information
	To supplement the patient’s voice
	To respect and prioritize the spouse
Take notes	To retain information
	To inform care and discussions with the patient after the appointment
* Express emotional support	** To protect personhood
	** To provide reassurance to reduce distress
* Promote patient engagement	** To protect personhood
	To provide a detailed narrative
**After the appointment**	
Debrief together	To cope with the disease together and be on the same page
	To clarify information from the appointment
* Engage in a relational ritual	** To re-focus on a positive, relational experience
**Ongoing or between appointments**	
Advocate for care	To coordinate care
	To ensure quality care

Note: Strategies and goals that are novel to the C^3^PM-Dementia model are indicated by single (*) and double asterisks (**), respectively.

## Data Availability

This qualitative study was not pre-registered. Access to de-identified data may be available upon request and with IRB approval from the senior author and funder.

## Abbreviations

The following abbreviations are used in this manuscript:

C^3^PM	Caregiver Clinical Communication Model
AD/ADRDs	Alzheimer’s disease or related dementias
